# Colorimetric Biosensor Based on Magnetic Enzyme and Gold Nanorods for Visual Detection of Fish Freshness

**DOI:** 10.3390/bios12020135

**Published:** 2022-02-21

**Authors:** Xia Xu, Xiaotian Wu, Shunqian Zhuang, Yucong Zhang, Yuting Ding, Xuxia Zhou

**Affiliations:** 1 College of Food Science and Technology, Zhejiang University of Technology, Hangzhou 310014, China; xuxia@zjut.edu.cn (X.X.); 17816879411@163.com (X.W.); zhuangsq337@163.com (S.Z.); zyc1981103570@163.com (Y.Z.); dingyt@zjut.edu.cn (Y.D.); 2 Key Laboratory of Marine Fishery Resources Exploitment & Utilization of Zhejiang Province, Hangzhou 310014, China; 3 National R&D Branch Center for Pelagic Aquatic Products Processing (Hangzhou), Hangzhou 310014, China; 4 Ninghai ZJUT Academy of Science and Technology, Ninghai 315600, China

**Keywords:** histamine, colorimetric biosensor, magnetic diamine oxidase, etching of gold nanorods, fish freshness

## Abstract

Histamine, an important safety index for aquatic products, can also be used as a freshness indicator for red-fleshed fish. In this work, magnetic graphene oxide (Fe_3_O_4_@GO, MGO) was applied to immobilize diamine oxidase (DAO) through a method of adsorption and covalent bonding. Under the optimized conditions, magnetic DAO prepared by adsorption immobilization had a higher enzyme activity than that of free enzyme, which was selected for the sensor construction. A colorimetric biosensor based on magnetic DAO induced etching of gold nanorods (AuNRs) was developed for the detection of histamine in fish. The developed biosensor showed an excellent response toward histamine with a low detection limit of 1.23 μM and had negligible interference from other diamines. With increasing the histamine concentration, the AuNRs after the reaction exhibited colors ranging from dark green to blue-green, blue, purple, red, and colorless. The etching induced multicolor change of AuNRs indicated the presence of different contents of histamine in mackerel during storage, and was consistent with the overall change in the content of the total volatile basic nitrogen (TVB-N). Thus, it was indicated that the proposed colorimetric biosensor with a naked-eye-detectable readout has a great potential to evaluate the freshness of red-fleshed fish high in histamine.

## 1. Introduction

Histamine is a kind of biological amine produced from histidine under the action of histidine decarboxylase after the death of a fish. It is the most abundant and main biogenic amine in red-fleshed fish mainly belonging to the family *Scombridae*, such as mackerel and tuna [1]. Histamine is also an important biomarker for food spoilage and can be used as a freshness index in this kind of fish [2,3]. In addition, the Food and Drug Administration (FDA) has stated that if the histamine content in fish exceeds 200 ppm, it can cause histamine poisoning and endanger the health of consumers [4]. Conventional detection methods for histamine are mainly chromatographic techniques such as capillary electrophoresis, gas chromatography, and high-performance liquid chromatography [5,6], which have limitations such as having time-consuming procedures, high costs, complicated operating procedures, and the need for professionals, and, therefore, such methods cannot meet the actual needs. Thus, the establishment of a fast, simple, and sensitive detection method for histamine that can effectively evaluate the freshness of red-fleshed fish has important practical significance and is challenging.

Optical biosensors with the advantages of being direct, real-time, and having label-free detection, make them ideal for histamine analysis, including colorimetric, chemiluminescent, fluorescent methods, and so on [7]. Therein, colorimetric biosensors based on the etching of gold nanorods (AuNRs) have received much attention in recent years [8,9]. The longitudinal absorption peak of AuNRs shifts because of the etching-induced decrease in the aspect ratio of nanorods often accompanied with a visible color change. Multicolor readout can also be realized towards visual quantification of the analytes by the naked eye. Diamine oxidase (DAO), which plays a key role in nitrogen metabolism, can be used as a recognition element of biosensors for histamine detection [10]. However, previous colorimetric biosensor based on the enzymatic etching of AuNRs cannot be reused, because it usually employs free enzymes as biosensing elements, and such elements are not easy to store [11]. In addition, the sensitivity of these methods is relatively low, which leads to a decrease in the potential of these methods for on-site detection applications.

Immobilized enzymes refer to a state in which water-soluble enzymes are treated by physical or chemical methods to make them insoluble in water, but still have an enzyme activity. The immobilized enzyme not only has a catalytic activity and specificity, but also improves the stability of the enzyme to pH and temperature. In addition, the immobilized enzyme can simplify the production process, and it can be separated from the reaction product by certain means, which not only enables the reuse of the enzyme, but also reduces the difficulty of product separation and purification [12]. The current commonly used immobilization methods can be divided into physical methods such as adsorption and embedding, and chemical methods such as covalent bonding and cross-linking [13,14,15]. Physical methods result in little loss of the enzyme activity, but there are problems of enzyme desorption and leakage; chemical methods use covalent bonds to immobilize enzymes, which increase the stability but lead to a loss of enzyme activity [16].

Magnetic graphene oxide (Fe_3_O_4_@GO, MGO) is a new type of enzyme immobilization carrier that not only has the magnetic characteristics of magnetic materials, but also includes the advantages of graphene oxide, such as a large specific surface area and a large number of oxygen-containing groups [17]. It can provide abundant binding sites for enzyme immobilization without modification. A magnet can be used to separate the reaction product from the immobilized enzyme, which solves the problem that GO is not easy to separate from the aqueous solution as an immobilized carrier [17]. Xie et al. [18] immobilized lipase on MGO by covalent bonding and used the immobilized lipase to produce biodiesel. The yield was as high as 92.8% and after five times of use, there was no significant loss of enzyme activity. Li et al. [19] proposed a special MGO with a three-dimensional network, which could effectively prevent the aggregation of GO. When it was used as an immobilized carrier for porcine pancreatic lipase, the immobilization efficiency reached 91% and the activity reached 90% of the free enzyme. After 10 cycles of repeated use, the enzyme activity of the immobilized enzyme was higher than 80% of the original enzyme activity.

In this study, MGO was applied as the support for the immobilization of DAO. Two enzyme immobilization methods, adsorption and covalent bonding, were selected and optimized to immobilize DAO. The magnetic DAO with a better performance was selected to construct a colorimetric biosensor for histamine, which could be easily separated from the solution by a magnet after enzymatic reaction. The etching of AuNRs occurred with the increasing histamine concentration, inducing a multicolor readout. The proposed biosensor was expected to evaluate the freshness of the red-fleshed fish by the naked eye according to the relationship between the histamine content and the total volatile basic nitrogen (TVB-N) value as the freshness indicator.

## 2. Materials and Methods

### 2.1. Chemical Reagents

1-Ethyl-3-(3-dimethylaminopropyl) carbodiimide (EDC) (no. E7750), N-hydroxysuccinimide (NHS) (no. 130672), DAO (D7876), histamine dihydrochloride (no. 53300), hydrochloride of tyramine, tryptamine and 2-phenylethylamine, dihydrochloride of putrescine and cadaverine, spermine tetrahydrochloride, and spermidine trihydrochloride were purchased from Sigma-Aldrich (St. Louis, MO, USA). l-histidine was purchased from Aladdin Industrial, Inc. (Shanghai, China). Horseradish peroxidase (HRP) was purchased from Sangon Biotech Co., Ltd. (Shanghai, China). The other reagents used in this study were analytical reagents from Sinopharm Chemical Reagent Co., Ltd. (Shanghai, China). MGO was obtained from Nanjing XFNANO Materials Tech Co., Ltd. (Nanjing, China). Ultrapure water was obtained from an HYJD ultrapure water system (Hangzhou, China).

### 2.2. DAO Immobilization

Preparation of the immobilized enzyme by adsorption [20] was as follows: 100 μL of DAO solution (100 mg/mL) was added to 1 mL of a MGO dispersion (10 mg/mL) and shaken at 37 °C for 2 h. After the reaction, the mixed material was adsorbed with an external permanent magnet. The precipitate was washed three times with 100 mM PB buffer (pH 5.9), and it was diluted to 5 mL. The supernatant and washing solutions were collected to measure the amount of residual DAO. The lower layer included the target product, MGO-A-DAO, and it was stored at 4 °C.

Preparation of the immobilized enzyme by covalent bonding was as follows [21]: 15 mg of NHS and 25 mg of EDC were mixed with 1 mL of the MGO dispersion (10 mg/mL), and then shaken at room temperature for 2 h to activate the carboxyl groups in MGO. Next, the excess NHS and EDC were removed through the addition of a magnet, and 1 mL of PB buffer (100 mM, pH 5.9) was added and ultrasonicated for 1 min; then, 100 μL of DAO solution (100 mg/mL) was added and shaken at 37 °C for 2 h. The remaining operations were the same as those described above in the adsorption method, and they led to the product, MGO-C-DAO.

### 2.3. Characterization of DAO Immobilization

Characterization of MGO was achieved through scanning electron (SEM) microscopy (Gemini 500, Carl Zeiss, Jena, Germany) and a 120-kV transmission electron (TEM) microscopy (Hitachi ht7700, Hitachi Ltd., Tokyo, Japan). DAO immobilization was characterized by X-ray diffraction (X’Pert Pro MPD, Philips, Eindhoven, Netherlands), a vibrating sample magnetometer (Versalab, Quantum Design, San Diego, CA, USA), and Fourier transform infrared spectroscopy (Nicolet 6700, Thermo Electron, Madison, WI, USA).

### 2.4. Performance Determination of MGO-DAO

#### 2.4.1. Enzyme Loading for MGO-DAO

The enzyme loading was determined using Coomassie Brilliant Blue [22], and the calculation of enzyme load is shown in Equation (1):

(1) $$\mathrm{Enzyme}\;\mathrm{load}\;(\mathrm{mg}/g)=\frac{C_{1}V_{1}-C_{2}V_{2}}{C_{3}V_{3}}\times 100$$

C_1_ is the protein concentration of the initial enzyme solution (mg/mL), V_1_ is the added amount of the initial enzyme solution (mL), C_2_ is the protein concentration in the collected supernatant and washing solutions (mg/mL), V_2_ is the volume of supernatant (mL), C_3_ is the concentration of MGO (mg/mL), and V_3_ is the volume of MGO (mL).

#### 2.4.2. Activity of MGO-DAO

The activity assay of magnetic DAO was proposed by Aarsen et al. [23] with certain modifications. The enzyme activity was obtained with the following equation. The DAO enzyme activity U is defined in this paper as 1 U oxidizing 1 μmol of histamine per hour at 37 °C.

(2) $$\mathrm{Enzyme}\;\mathrm{activity}\;(U/10\;\mathrm{mg}\;\mathrm{MGO})=\frac{A_{440}\times 1\times 6}{11.3}$$

A_440_ is the absorbance value at a wavelength of 440 nm, 1 is the total solution volume (mL), 6 is the conversion factor from U/10 min to U/h, and 11.3 is the extinction coefficient of DAO at 440 nm.

### 2.5. Preparation of AuNRs

AuNRs were synthesized through the seed-mediated growth method [24]. First, 0.6 mL of 0.01 M ice-cold NaBH_4_ was added to 10 mL of a mixture of 0.1 M CTAB and 0.25 mM HAuCl_4_ under vigorous stirring. The seed solution, after 2 min of stirring, was kept at room temperature for 2 h. Then, 100 mL of 0.2 M CTAB and 3 mL of 4 mM AgNO_3_ were added to 100 mL of 1 mM HAuCl_4_, which was then mixed with 1.58 mL of 79 mM ascorbic acid under gentle stirring. Finally, 0.24 mL of seed solution was added, and the solution was kept at 30 °C for 2 h. The prepared AuNRs were purified through centrifugation and stored in deionized water. The absorption spectra of AuNRs were measured from 400–900 nm on a UV-6100s spectrometer (Mapada, Shanghai, China).

### 2.6. Histamine Detection with MGO-DAO and AuNRs

After the supernatant was removed from the MGO-A-DAO solution, 150 μL of PB buffer (20 mM, pH 5.9), 800 μL of different concentrations of histamine (0, 5, 10, 20, 30, 40, 50, 60, 70, 80, 90, 100, 120, 140, 160, 180, 200, and 400 μM), 20 μL of KI (40 mM), and 10 μL of Na_2_MoO_4_ (1 mM) were added and then shaken at 55 °C for 30 min. After the enzyme-catalyzed reaction was completed, a magnet was used to separate the layers and transfer the supernatant to another clean 1.5 mL centrifuge tube (the bottom layer, which included MGO-A-DAO, was redissolved and stored in a PB buffer). Then, 100 μL of the prepared AuNRs and 20 μL of HCl (2 M) were added into the supernatant and kept at 55 °C for 20 min. The absorption spectra of the resulting solutions were measured and photos were taken to record the change in the solution color reacted with different concentrations of histamine.

The specificity of the proposed method was evaluated with the same detection procedure as described above. l-histidine and seven common biogenic amines (e.g., putrescine and cadaverine) as alternatives of histamine were used for detection with a concentration of 160 μM.

### 2.7. Detection of Histamine in Fish Samples

Mackerel, as the test sample, was purchased from Zhoushan, China. The fish was frozen very shortly after being caught, transported to the laboratory under the cold chain as soon as possible, and stored at −18 °C. After the skin, spines, and blood of the mackerel were cleaned, 5 g of the homogenized mackerel meat was dispersed in 65 mL of 5% trichloroacetic acid, followed by ultrasonic extraction for 30 min. After centrifugation at 5000 r/min for 10 min, the supernatant was taken, and the pH was adjusted to neutral to obtain the extract. The proposed colorimetric biosensor based on MGO-DAO and AuNRs was used to determine the histamine content in the extract. The accuracy of the proposed method was evaluated by HPLC [25]. The determination of TVB-N content in mackerel referred to the Chinese national standard (GB 5009.228-2016).

## 3. Results and Discussion

### 3.1. Characterization of the DAO Immobilization

**Figure 1:**
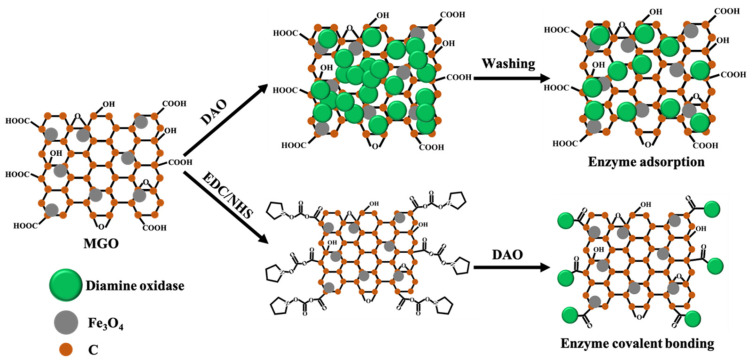
Schematic description of DAO immobilization on MGO.

The strategy for immobilizing DAO is shown in Figure 1. The adsorption method uses physical adsorption, especially electrostatic adsorption, to immobilize DAO on the MGO [26]. The covalent bonding method activates the -COOH group of MGO through EDC/NHS, and then the -COOH group combines with the -NH_2_ group of DAO to form a covalent bond and immobilize on the MGO [27].

According to the TEM and SEM images of the MGO nanocomposite (shown in Figure S1), as expected, a large amount of Fe_3_O_4_ nanoparticles (about 10 nm in diameter) were anchored on the surface of the GO sheet structure. The characterization results of DAO before and after immobilization are shown in Figure S2. In the FTIR spectra of MGO-DAO, there were not only the characteristic peak of 548 cm^−1^ belonging to Fe-O, but also peaks at 1628 cm^−1^ and 1531 cm^−1^, which indicated the presence of the -CO-NH- group of DAO [18,21]. DAO was shown to be successfully immobilized on the surface of the MGO. However, the signal of MGO-DAO was low, mainly because the enzyme loaded was too small. The curves of MGO and MGO-DAO in the XRD figure had six obvious diffraction peaks belonging to Fe_3_O_4_ at 30.05°, 35.60°, 43.20°, 53.60°, 57.15°, and 62.60° [28], indicating that the immobilization of DAO would not destroy the crystal form of Fe_3_O_4_ magnetic nanoparticles on MGO. The saturation magnetization of MGO-C-DAO and MGO-A-DAO evaluated by VSM decreased from the initial MGO of 46.60 emu/g to 40.03 and 36.27 emu/g, respectively. This may be due to the decline in the Fe_3_O_4_ proportion in the composite because of the binding of DAO [29]. The three hysteresis loops were all close to the origin, which demonstrated that the MGO had good superparamagnetism before and after immobilization with DAO. MGO-DAO could be easily separated from the solution by a magnet, indicating the successful preparation of magnetic DAO.

### 3.2. Optimization of the DAO Immobilization

**Figure 2:**
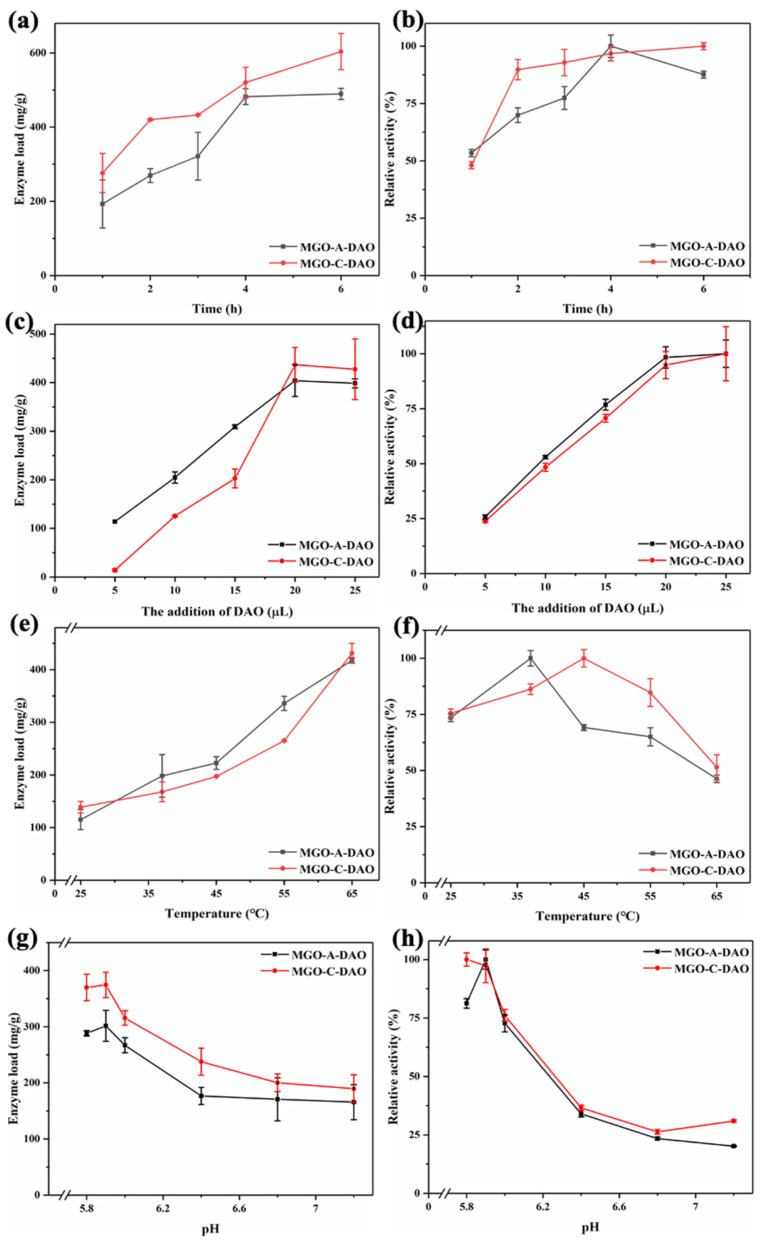
The effect of enzyme immobilization conditions on the immobilization effect of DAO (immobilization time (**a**,**b**), enzyme addition (**c**,**d**), immobilization temperature (**e**,**f**), immobilization pH (**g**,**h**). Among them, (**a**,**c**,**e**,**g**) are the enzyme loading and (**b**,**d**,**f**,**h**) are the relative enzyme activity (the highest value is 100%)).

As shown in Figure 2a,b, the enzyme loading and relative enzyme activity of MGO-A-DAO initially increased as the immobilization time increased, and then reached a maximum at 4 h. The possible reason was that the MGO material could not provide more binding sites for DAO. As the immobilization time was further extended, the steric hindrance of the enzyme increased and the enzyme activity decreased [30]. Therefore, the optimal immobilization time of MGO-A-DAO was 4 h. In contrast, the enzyme loading capacity of MGO-C-DAO increased with the extension of the immobilization time in this study, because there were many -COOH groups on the surface of MGO, providing enough binding sites for enzyme immobilization. However, its relative enzyme activity did not change much within the immobilization time from 2 to 6 h, and only increased by 11%. Therefore, 2 h was selected as the optimal immobilization time for MGO-C-DAO.

As the additional amount of DAO (100 mg/mL) increased from 5 to 20 μL, the enzyme loading and relative activity of MGO-DAO increased and then reached a maximum (Figure 2c,d). When the addition of DAO exceeded 20 μL, the enzyme loading and activity of MGO-DAO reached a steady state. This is because the binding sites on the surface of MGO were limited, and only a certain amount of DAO could be immobilized on MGO.

The enzyme loading increased with increasing the immobilization temperature from 25 to 65 °C, while the relative activity of MGO-DAO showed a trend of first increasing and then decreasing (Figure 2e,f). The increase in temperature could help MGO to bind more enzymes. However, a high temperature could destroy the active site of the enzyme at the same time, leading to a decrease in the enzyme activity, which is consistent with the research results of Liu et al. [31]. The optimal temperatures for the preparation of MGO-A-DAO and MGO-C-DAO were 37 °C and 45 °C, respectively.

pH is an important parameter that affects the enzyme activity of MGO-DAO [32]. Figure 2g,h shows that the optimal pH of the adsorption method was 5.9, which was consistent with the optimal pH for the decomposition of histamine catalyzed by free DAO [11,33]. Only when the pH was lower than 6.0 could electrostatic adsorption be achieved in order to immobilize a large amount of DAO on MGO [26]. The optimal pH range of the covalent bonding method was 5.8–5.9, at which the enzyme loading and activity of MGO-C-DAO reached the maximum. A further increase in pH would affect the active site of DAO to be affected, thereby reducing the ability of DAO to catalyze histamine.

### 3.3. Reusability of the MGO-DAO

**Figure 3:**
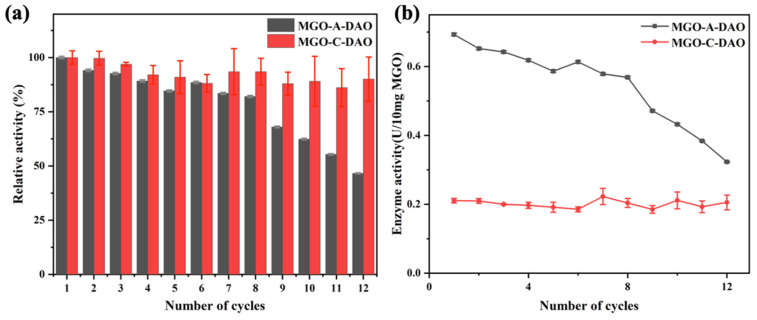
Reusability of MGO-DAO (**a**) relative enzyme activity (take the first enzyme activity as 100%) and (**b**) enzyme activity.

The reusability of the magnetic DAO was tested before it was applied to the biosensor (Figure 3). MGO-A-DAO maintained more than 80% of the original enzyme activity after eight times of repeated use. As the number of repeated uses increased, some enzymes lost their activity, and some enzymes were desorbed from the MGO [34]. In contrast, MGO-C-DAO still maintained more than 85% of the original enzyme activity after 12 times of use. However, as shown in Figure 3b, the enzyme activity of MGO-A-DAO after 12 times of repeated uses was 0.32 U/10 mg MGO, which was still much greater than the initial enzyme activity of MGO-C-DAO. The enzyme activity of optimized MGO-A-DAO was more than 120% that of the free DAO (Table S1). It was probably due to the two-dimensional network structure of graphene, the contact between the enzyme, and the substrate increased, exposing more enzyme active sites, thus improving the catalytic activity of DAO toward histamine [35]. Although MGO-C-DAO had better reusability and stability (Figure 3 and Figure S3), immobilization through covalent bonding caused greater damage to the enzyme activity. Therefore, we finally immobilized DAO on MGO through the optimized adsorption method and applied it to the subsequent colorimetric biosensor.

### 3.4. Principle of the Colorimetric Biosensor for Fish Freshness

**Figure 4:**
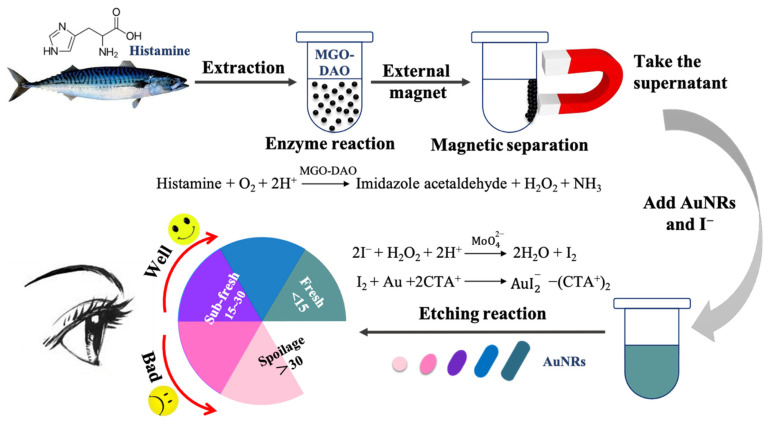
Schematic diagram of the colorimetric biosensor based on magnetic DAO and AuNRs for fish freshness, including enzyme reaction and etching reaction; the designed freshness color label judged by the TVB-N range (100 mg/100 g).

Figure 4 shows a schematic diagram of the colorimetric biosensor based on magnetic DAO and AuNRs for the detection of histamine in fish, and the evaluation of fish freshness. The characterization of the synthesized AuNRs is shown in Figure S4, with a longitudinal adsorption peak of 658 nm, and average length and width of 57 and 28 nm, respectively. As shown in the schematic diagram, the histamine extracted from the fish samples was decomposed under the catalysis of MGO-DAO to produce H_2_O_2_. Then, under the intervention of Na_2_MoO_4_ which has a peroxidase-like activity, I^−^ could be quickly reduced to a strong reducing agent of I_2_ by H_2_O_2_, which could etch AuNRs longitudinally. This would lead to a blueshift in the longitudinal adsorption peak of AuNRs, companied by a series of color changes in the solution. The higher the histamine content, the more obvious the etching of AuNRs and the greater the longitudinal peak blueshift.

The concentration of the enzyme in the solution was of great significance to the etching reaction of AuNRs [8]. A high concentration of DAO in the solution inhibited the occurrence of the etching reaction of AuNRs, while a low concentration of DAO led to less production of H_2_O_2_ and thereby a low sensitivity for histamine detection [11]. The use of MGO-DAO in the colorimetric biosensor could solve this problem completely. MGO-DAO had strong magnetic properties and could be easily separated from the solution by a magnet after the enzymatic reaction, reducing the protein content in the solution and enabling the etching reaction to occur smoothly. Histamine is used as a freshness index in red-fleshed fish. Thus, the designed colorimetric biosensor could be applied for evaluation of fish freshness.

### 3.5. Optimization of the Detection Conditions

Effect of different detection conditions on the longitudinal peak blueshift (Δλ) of AuNRs were studied in this paper, with a histamine concentration of 100 μM. It can be seen from Figure S5a that the Δλ of AuNRs increased with the temperature of etching reaction rising from 25 to 65 °C; when the temperature was higher than 50 °C, the change of Δλ was not obvious with the increase of temperature. In order to unify the temperature of the whole sensing process, the temperature of the etching reaction was adjusted to 55 °C, consistent with that of the enzyme reaction. Figure S5b shows that the Δλ of AuNRs reached a maximum when the concentration of KI was increased to 40 mM. As shown in Figure S5c,d, the optimal time for the enzyme reaction and the etching reaction was 30 and 20 min, respectively; the increase of Δλ was not significant when the time for the two reactions exceeded 30 and 20 min, respectively. Figure S5e shows that the optimal addition amount of HCl (2 M) was 20 μL, and more or less addition would reduce the etching of AuNRs.

### 3.6. Detection of Histamine with the Colorimetric Biosensor

**Figure 5:**
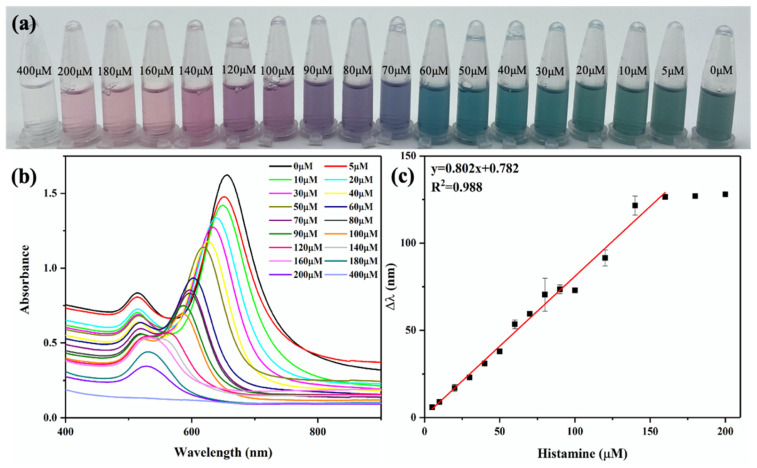
Histamine detection curve: (**a**) color change picture; (**b**) UV−VIS absorption spectrum (400–900 nm); (**c**) standard curve for histamine detection.

It can be seen from Figure 5a that when the concentration of histamine increased from 0 to 400 μM, the color of AuNRs showed a series of gradient changes from dark gray to blue-green, blue, purple, red, and colorless. The color change of the AuNR solution could be easily observed by the naked eye in order to achieve semiquantitative visual detection of histamine. As shown in Figure S6, AuNRs etched along the longitudinal axis with the increase in histamine concentration, and the shape changed from nanorods to nanospheres. In addition, the longitudinal peak of the AuNRs also gradually blueshifted to a lower band with the increasing histamine concentration, until it overlapped with the transverse adsorption peak (Figure 5b).

The color change and the blueshift of the longitudinal peak of AuNRs were directly proportional to the concentration of histamine. Figure 5c shows the linear relationship between the longitudinal peak blueshift distance Δλ of the AuNRs and the histamine concentration. The proposed regression equation for detecting the histamine content can be expressed as y = 0.802x + 0.782; this equation shows a highly linear relationship (R^2^ = 0.988) in the histamine concentration range of 5–160 μM. According to the rule of 3S/N (S is the standard deviation of the intercept and N is the slope of the standard curve), the calculated limit of detection (LOD) was 1.23 μM, which was only 1/16 of the free enzyme [11]. Its detection range and LOD were compared with those of previously reported biosensors for histamine detection, and the results are presented in Table 1. The results demonstrated that this proposed colorimetric biosensor with magnetic DAO and AuNRs had a good sensitivity, and the signal readout was simpler and more convenient.

**Figure 6:**
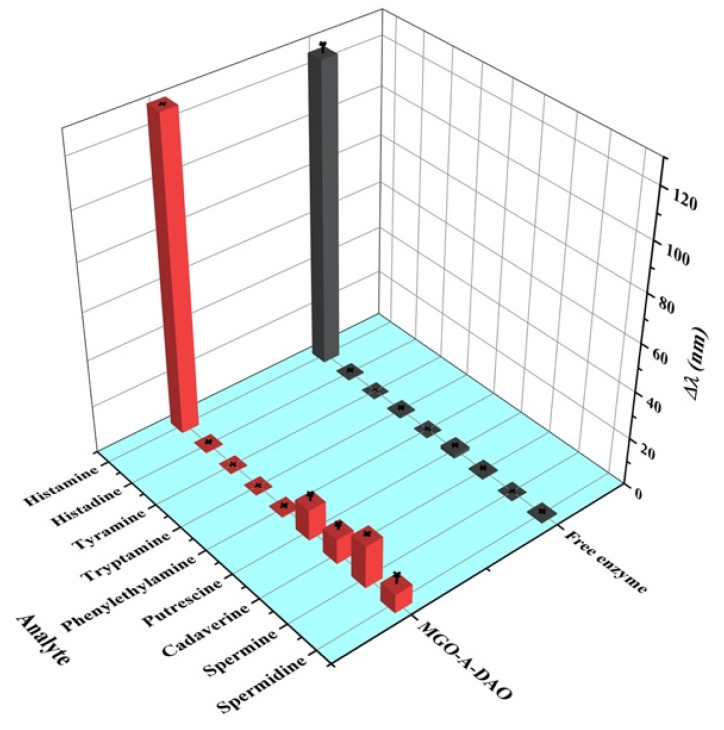
Specificity evaluation of the fabricated biosensor: Δλ of AuNRs responding to histamine and other common analytes at a concentration of 160 μM.

Specificity is one of the important parameters for evaluating sensor performance. In this paper, the response values of the colorimetric biosensor with MGO-A-DAO and free DAO to l-histidine and common biogenic amines were compared (Figure 6). Under the optimal reaction conditions, free DAO had extremely low response values to these high-concentration histamine analogs (160 μM), showing its excellent specificity. In contrast, the proposed biosensor fabricated from MGO-A-DAO had a certain response to high concentrations of putrescine, cadaverine, spermine, and spermidine. The response value of spermine was 13.8% of the histamine response value, and the response value of other diamines was less than 10%. The reason was speculated to be because the ability of DAO to catalyze other diamines was also improved after immobilization on the support material, MGO. In addition, real fish samples after extraction and dilution hardly contained such a high content of diamine (160 μM), which did not reach the concentration that produced the sensing response. Therefore, the interference signal had a negligible effect on the actual detection results, and the colorimetric biosensor for histamine had a good specificity.

To verify the accuracy of the proposed colorimetric biosensor, HPLC was used for comparison to detect the histamine concentration of the spiked fish sample. The spiked concentrations of histamine in mackerel samples were 25, 50, and 100 μM, respectively. As shown in Table 2, the detection recovery rate of the colorimetric biosensor based on magnetic DAO and AuNRs was between 92.70% and 96.20%, and the RSD was between 1.57% and 5.42%. There was no significant difference between the detection results of the proposed biosensor and the HPLC method, indicating that the sensor could be applied for the detection of histamine in real fish samples.

### 3.7. Evaluation of Fish Freshness

**Figure 7:**
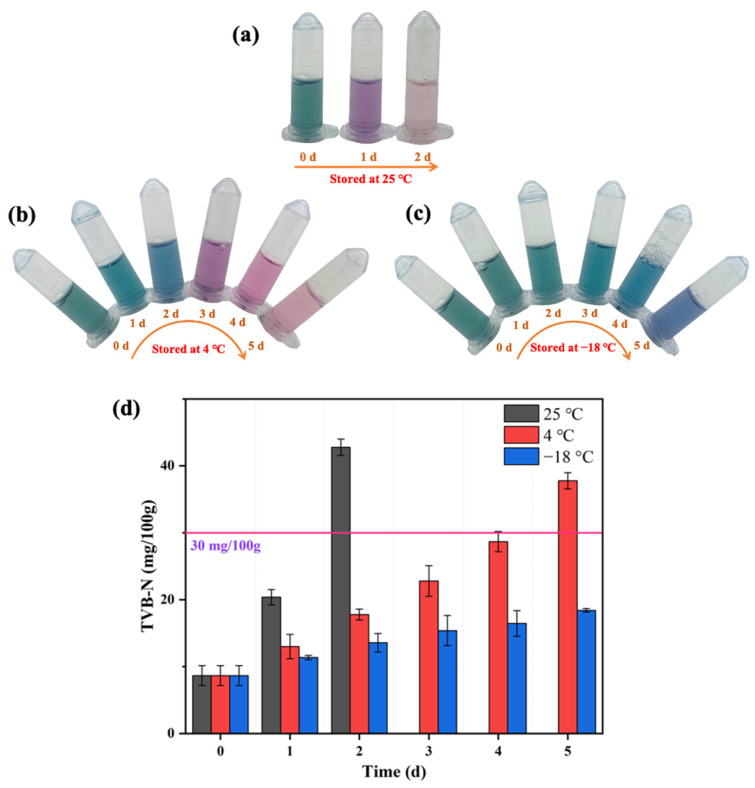
Evaluation of the freshness of mackerel stored at 25 °C (**a**), 4 °C (**b**), and −18 °C (**c**) by the proposed biosensor and changes of TVB-N content of mackerel stored at different temperatures (**d**).

The TVB-N content of mackerel stored at 25 °C, 4 °C, and −18 °C was determined to construct the relationship between the sensor response and mackerel freshness (Figure 7d). The initial TVB-N of the fresh mackerel was 8.66 mg/100 g. After two days of storage at 25 °C, the TVB-N of mackerel surged to 42.78 mg/100 g and the color of AuNRs after etching in this biosensor was light red (Figure 7a), and the fish body appeared to have an obvious putrefaction odor. The TVB-N of the mackerel samples stored at 4 °C increased with the storage time, and the corresponding AuNRs exhibited a range of colors from initially dark green to blue-green (the first day), light blue (the second day), purple (the third day), red (the fourth day), and light red (the fifth day; Figure 7b). When the color of the AuNRs was red or light red, the mackerel had deteriorated, with the TVB-N content of mackerel exceeding the limit of 30 mg/100 g. The TVB-N of the mackerel stored at −18 °C for 5 days was 18.42 mg/100 g, and the AuNRs turned from dark gray to dark blue (Figure 7c). Therefore, according to the changes of TVB-N in mackerel during storage, the color of AuNRs after the etching reaction was divided into several grades to reflect their freshness (Figure 4). The AuNRs exhibiting dark gray or blue-green colors after the reaction indicated that the fish sample was fresh. Blue, purple, or purplish red colors indicated that the fish were sub-fresh. Red and a red color that gradually faded away represented the spoilage of fish. Therefore, the proposed colorimetric biosensor has great potential for the freshness evaluation of red-fleshed fish by the naked eye. However, it is worth noting that there may be differences in the relationship between histamine and TVB-N content in different types of fish. Therefore, further research is still needed to expand the application of the novel colorimetric biosensor in more kinds of fish.

## 4. Conclusions

A colorimetric biosensor for fish freshness based on histamine content was proposed using magnetic DAO as a biosensing element and etched AuNRs as signal converters. The reusable magnetic DAO prepared by immobilizing DAO on MGO through an adsorption method after optimization not only improved the separation efficiency of the enzyme and reaction products, but it also improved the enzyme activity and the efficiency of the AuNR etching reaction. The developed colorimetric biosensor showed an excellent response to histamine with negligible interference, and the detection limit and range were 1.23 μM and 5–160 μM, respectively. As the concentration of histamine increased, the AuNRs after the etching reaction exhibited a range of colors from dark green to blue-green, blue, purple, red, and colorless, which could be correlated with the TVB-N value to reflect the change in the freshness of mackerel samples during storage. Overall, the designed colorimetric biosensor based on magnetic DAO and AuNRs has great potential to evaluate the freshness of red-fleshed fish high in histamine by the naked eye, and it has broad application prospects in the field of aquatic product quality detection.

## Figures and Tables

**Table 1:** Biosensors used in the detection of histamine.

Biosensing Element	Detection Method	Linear Range	LOD	Ref.
DAO	Colorimetric	5–160 μM	1.23 μM	This method
DAO	Amperometry	9–900 μM	8.46 μM	[36]
Antibody	Amperometry	56.25 µM–1.8 mM	30.7 μM	[37]
Aptamer	Impedance	1 μM–5 mM	4.83 mM	[38]
Aptamer	Fluorescence	1–100 μM	1 μM	[39]
DAO and horseradish peroxidase	Optical	0~1.5 mM	86 μM	[40]

**Table 2:** Recovery of spiked histamine in fish samples with the proposed biosensor and HPLC.

Sample	Spiked/μM	Detected/μM	Recovery/%	RSD/%	HPLC Detected/μM
1	25	26.28	96.20	5.42	27.23
2	50	54.42	95.84	4.22	56.50
3	100	98.95	92.70	1.57	106.28

## Supplementary Materials

The following supporting information can be downloaded at https://www.mdpi.com/article/10.3390/bios12020135/s1. Figure S1: TEM (a) and SEM (b) photos of MGO. Figure S2: FTIR (a), XRD (b), VSM (c) of DAO before and after immobilization on MGO; (d) shows the MGO-DAO and solution was easily separated by a magnet. Figure S3: Thermal stability (a) and pH stability (b) of free and immobilized DAO. Figure S4: TEM images, absorption spectrum and solution color of the synthesized AuNRs. Figure S5: Effect of different etching reaction temperature (a), KI concentration (b), enzyme reaction time (c), etching reaction time (d) and the addition of HCl (2 M) (e) on longitudinal peak blueshift of AuNRs. Figure S6: TEM images of AuNRs after reaction with the increasing concentration of histamine (From a and b (0 μM) to c and d (50 μM), e and f (200 μM)). Table S1: Enzyme activity of MGO-A-DAO, MGO-C-DAO, and free DAO.
