# Supplementary material for: Colorimetric Biosensor Based on Magnetic Enzyme and Gold Nanorods for Visual Detection of Fish Freshness
Source: Biosensors (Basel). 2022 Feb 21;12(2):135. doi: 10.3390/bios12020135 (PMC8870018; doi:10.3390/bios12020135)
Supplement: Supplementary file 1 [file biosensors-12-00135-s001.zip › biosensors-1595467-supplementary.pdf]

biosensors

Supplementary materials

Colorimetric Biosensor Based on Magnetic Enzyme and Gold Nanorods for Visual Detection of Fish Freshness

Xia Xu [1,2,3,4], Xiaotian Wu [1,2,3], Shunqian Zhuang [1], Yucong Zhang [1], Yuting Ding [1,2,3] and Xuxia Zhou [1,2,3,*]

1 College of Food Science and Technology, Zhejiang University of Technology, Hangzhou 310014, China; xuxia@zjut.edu.cn (X.X.); 17816879411@163.com (X.W.); zhuangsq337@163.com (S.Z.); zyc1981103570@163.com (Y.Z.); dingyt@zjut.edu.cn (Y.D.)
2 Key Laboratory of Marine Fishery Resources Exploitment & Utilization of Zhejiang Province, Hangzhou 310014, China
3 National R&D Branch Center for Pelagic Aquatic Products Processing (Hangzhou), Hangzhou 310014, China
4 Ninghai ZJUT Academy of Science and Technology, Ninghai 315600, China
* Correspondence: xzhou@zjut.edu.cn; Tel.: 86-571-88320237

Citation: Xu, X.; Wu, X.; Zhuang, S.; Zhang, Y.; Ding, Y.; Zhou, X. Colorimetric Biosensor Based on Magnetic Enzyme and Gold Nanorods for Visual Detection of Fish Freshness. *Biosensors* **2022**, *12*, 135. https://doi.org/10.3390/bios12020135

Received: 28 January 2022
Accepted: 20 February 2022
Published: 21 February 2022

Publisher's Note: MDPI stays neutral with regard to jurisdictional claims in published maps and institutional affiliations.

Copyright: © 2022 by the authors. Submitted for possible open access publication under the terms and conditions of the Creative Commons Attribution (CC BY) license (https://creativecommons.org/licenses/by/4.0/).

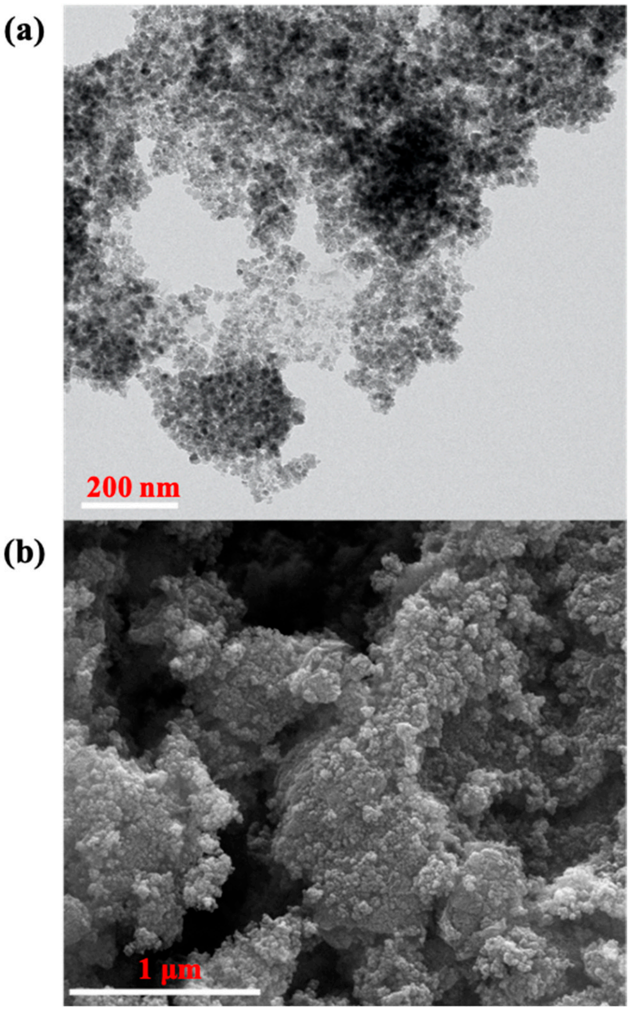

Figure S1. TEM (**a**) and SEM (**b**) photos of MGO.

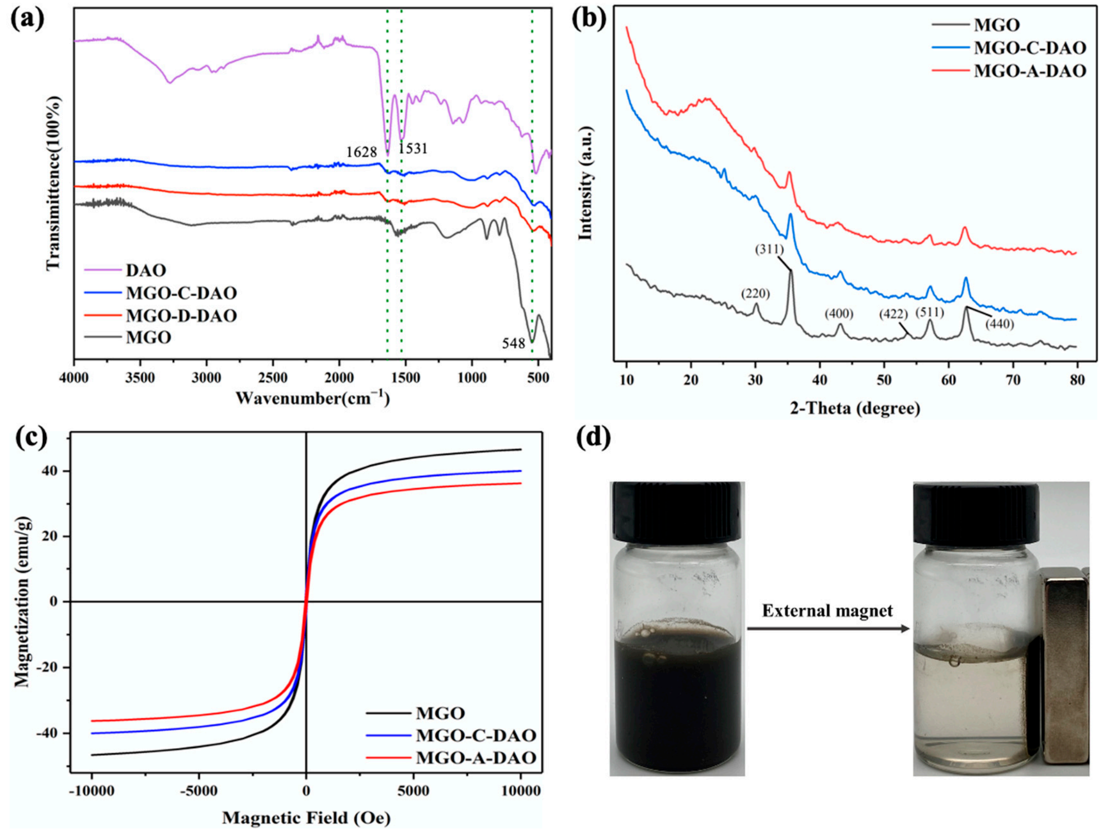

Figure S2. FTIR (**a**), XRD (**b**), VSM (**c**) of DAO before and after immobilization on MGO; (**d**) shows the MGO-DAO and solution was easily separated by a magnet.

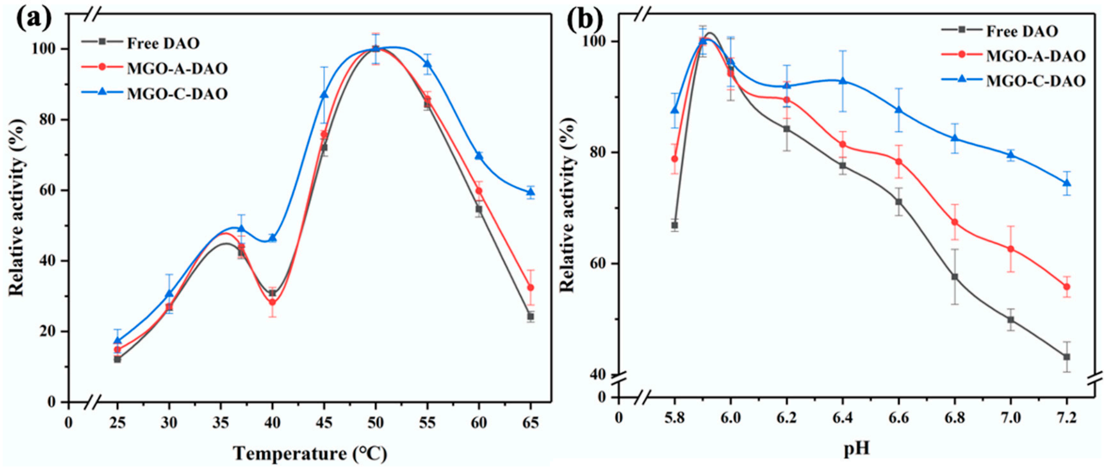

Figure S3. Thermal stability (**a**) and pH stability (**b**) of free and immobilized DAO.

Table S1. Enzyme activity of MGO-A-DAO, MGO-C-DAO, and free DAO.

Enzymes	Enzyme activity/ U/10mg MGO	Compared to the free enzyme
MGO-A-DAO	0.693	128.1%
MGO-C-DAO	0.211	39.0%
Free DAO	0.541	100.0%

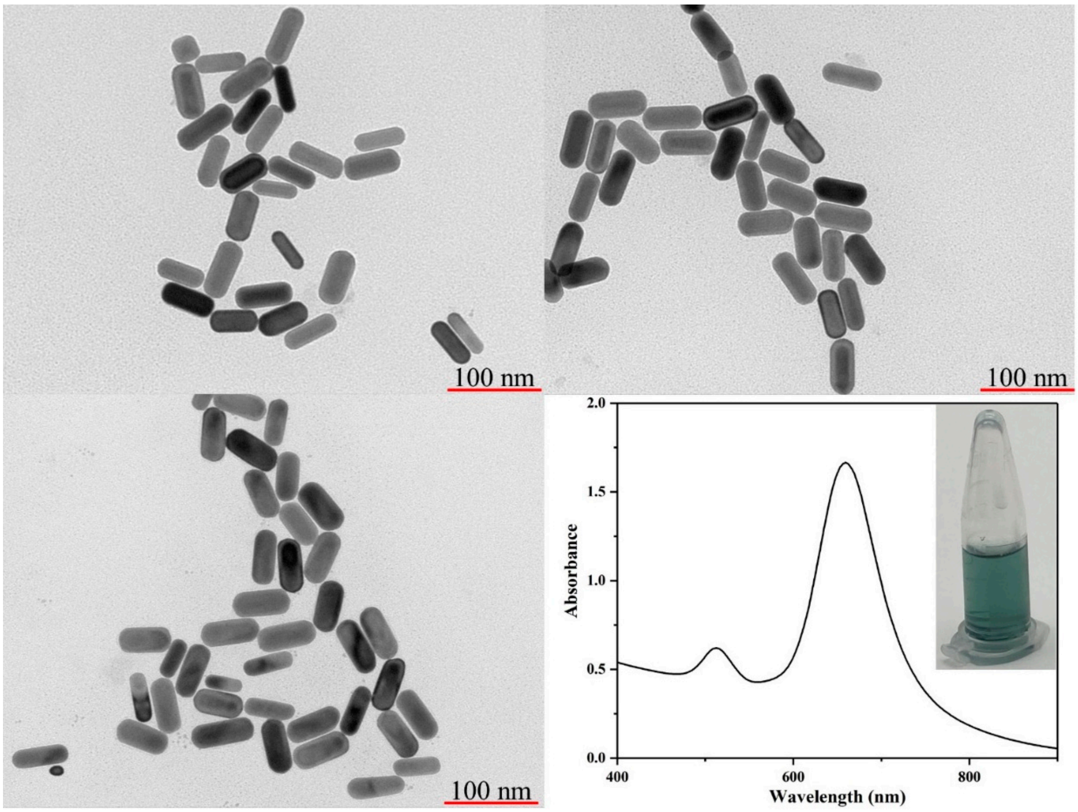

Figure S4. TEM images, absorption spectrum and solution color of the synthesized AuNRs.

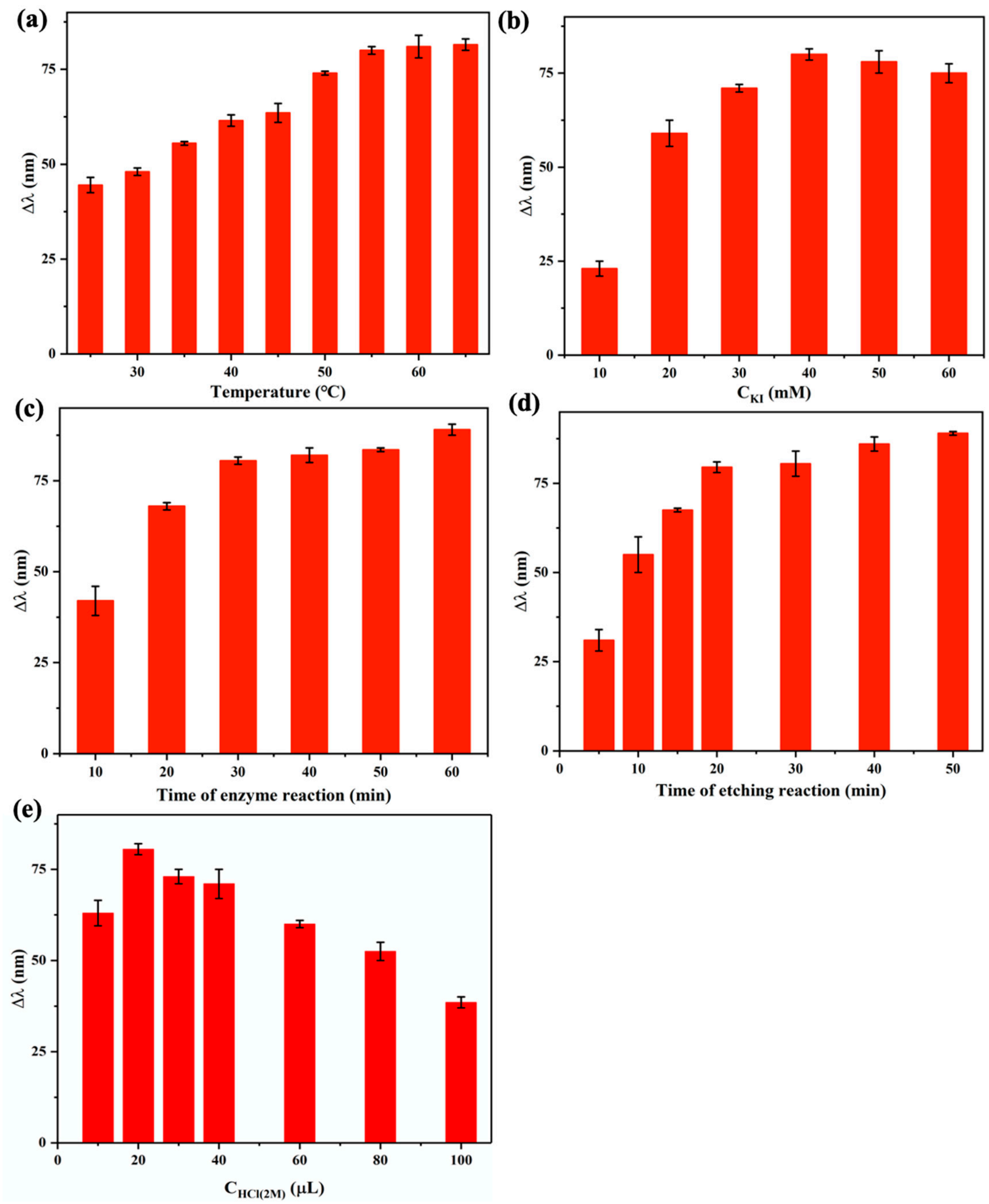

Figure S5. Effect of different etching reaction temperature **(a)** (enzyme reaction time 30 min, KI 40 mM, 2 M HCl 20 μL, etching time 30 min), KI concentration **(b)** (temperature 55℃, enzyme reaction time 30 min, 2 M HCl 20 μL, etching time 30 min), enzyme reaction time **(c)** (temperature 55℃, KI 40 mM, 2 M HCl 20 μL, etching time 30 min), etching reaction time **(d)** (temperature 55℃, KI 40 mM, enzyme reaction time 30 min, 2 M HCl 20 μL) and the addition of HCl (2 M) **(e)** (temperature 55℃, KI 40 mM, enzyme reaction time 30 min, etching time 20 min) on longitudinal peak blueshift of AuNRs. Optimized MGO-A-DAO and standard solution of histamine (100 μM) were used in the experiment.

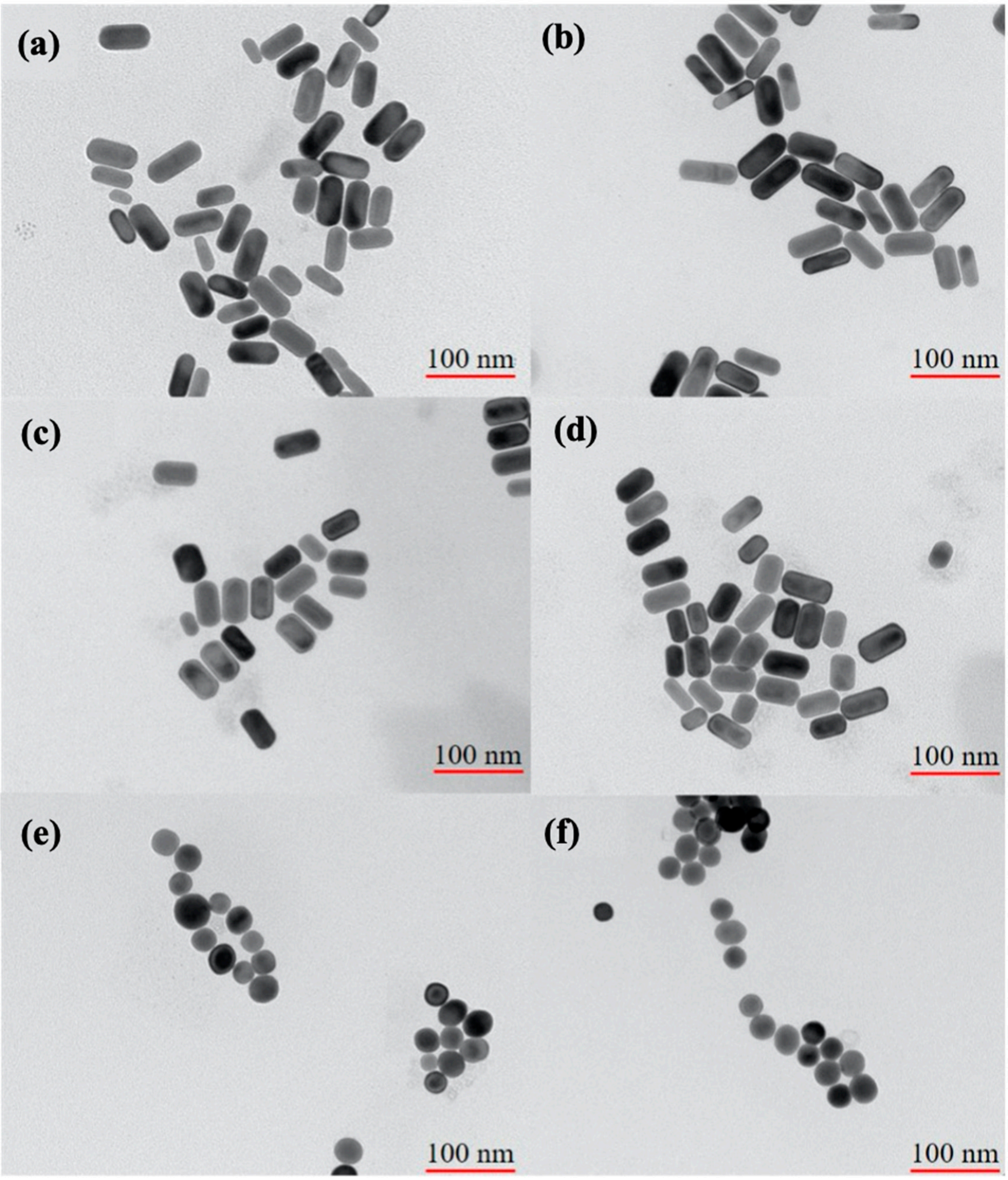

Figure S6. TEM images of AuNRs after reaction with the increasing concentration of histamine (From **(a)** and **(b)** (0 μM) to **(c)** and **(d)** (50 μM), **(e)** and **(f)** (200 μM)).
